# Prevalence and Clinical Significance of Gastric‐Type Mucosal Lesions in Non‐Ampullary Duodenal Epithelial Tumors Based on Mucin Phenotype

**DOI:** 10.1002/deo2.70443

**Published:** 2026-10-26

**Authors:** Kazuya Ohno, Eiji Nakatani, Takafumi Kurokami, Asami Kawai, Masanori Matsuda, Yuichi Masui, Tatsunori Satoh, Taiyo Hirata, Shodai Takeda, Yoshihiro Uehara, Kodai Baba, Yuka Ozaki, Tomoki Hikichi, Haruna Takahashi, Koki Sakaguchi, Kazuhito Honda, Tomomi Maeda, Moeri Hotta, Naofumi Nakamura, Makoto Suzuki, Ken Haruma

**Affiliations:** ^1^ Department of Gastroenterology Shizuoka General Hospital Shizuoka Japan; ^2^ Biostatistics and Health Data Science Graduate School of Medical Science Nagoya City University Aichi Japan; ^3^ Department of Pathology Shizuoka General Hospital Shizuoka Japan; ^4^ Department of General Internal Medicine 2 Kawasaki Medical School Okayama Japan

**Keywords:** endoscopic gastric mucosal atrophy, gastric‐type mucosal lesion, *Helicobacter pylori*, non‐ampullary duodenal epithelial tumor, smoking

## Abstract

**Objectives:**

Non‐ampullary duodenal epithelial tumors differ in clinicopathological features according to mucin phenotype. Gastric‐type tumors have distinct biological features; however, their associated mucosal background remains unclear. In this study, gastric foveolar metaplasia, gastric heterotopia, and Brunner's gland hyperplasia were collectively referred to as gastric‐type mucosal lesions (G‐MLs). We aimed to assess their prevalence and associations with mucin phenotype and clinicopathological factors.

**Methods:**

We retrospectively analyzed the data of 130 patients with 130 resected non‐ampullary duodenal epithelial tumors at a single center. Tumors were classified according to mucin phenotype. The prevalence of concomitant G‐MLs and associated factors was evaluated. Exploratory Firth‐penalized logistic regression analysis was used to assess the association between gastric‐type tumors and G‐ML presence.

**Results:**

Concomitant G‐MLs were identified in 44 patients (33.8%). Their prevalence was higher in gastric‐type tumors (13/16; 81.3%) than in gastric–intestinal‐type tumors (10/37; 27.0%) and intestinal‐type tumors (21/77; 27.3%). G‐MLs were associated with mild endoscopic gastric mucosal atrophy and smoking status and were inversely associated with current *Helicobacter pylori* infection. In the exploratory fully adjusted model, gastric‐type tumors remained positively associated with the presence of G‐MLs (adjusted odds ratio, 7.00; 95% confidence interval, 1.98–30.95; *p* = 0.002).

**Conclusions:**

G‐MLs were present in approximately one‐third of patients and were most frequent in gastric‐type tumors. These findings indicate an association between G‐MLs and gastric‐type tumors; however, the cross‐sectional design does not allow inference of a biological relationship.

**Trial Registration:**

N/A.

## Graphical abstract

Gastric‐type mucosal lesions are identified in 33.8% of patients with non‐ampullary duodenal epithelial tumors and are especially frequent in gastric‐phenotype tumors (81.3%). Their presence is associated with gastric mucosal atrophy and smoking, while current Helicobacter pylori infection shows an inverse association, suggesting a link between the duodenal mucosal background and tumor mucin phenotype.

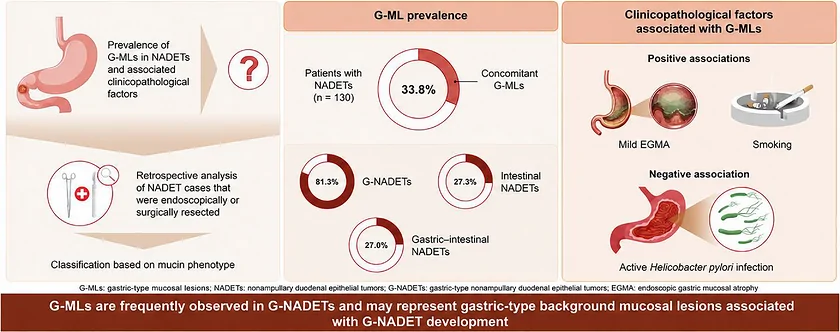

## 1 Introduction

Non‐ampullary duodenal epithelial tumors (NADETs) are relatively uncommon, but are being increasingly detected because of advances in endoscopic imaging and the widespread use of screening endoscopy [1]. NADETs are heterogeneous lesions with various clinicopathological features and malignant potential. The mucin phenotype is reportedly associated with the biological behavior of NADETs. Gastric‐type NADETs (G‐NADETs) are less common than intestinal‐type NADETs but are considered to have distinct clinicopathological features and a higher malignant potential [2].

Several gastric‐type mucosal lesions (G‐MLs), including gastric foveolar metaplasia, gastric heterotopia, and Brunner's gland hyperplasia, may be observed in the duodenum [3]. In the present study, these lesions were collectively referred to as G‐MLs because they share gastric‐type epithelial features. Matsubara et al. [4] reported activating mutations in GNAS and KRAS in gastric foveolar metaplasia, gastric heterotopia, and duodenal adenocarcinoma. However, the clinical association between G‐MLs and G‐NADETs has not been adequately investigated.

The pathogenesis of G‐NADETs remains unclear. We previously reported an association between G‐NADETs and mild endoscopic gastric mucosal atrophy (EGMA) [5]. However, the distribution of concomitant mucosal lesions in patients with G‐NADETs has not been fully characterized.

We hypothesized that the prevalence of concomitant G‐MLs differs according to the mucin phenotype of NADETs. Therefore, we aimed to assess the prevalence of G‐MLs in patients with NADETs and evaluate their association with the mucin phenotype of NADETs, as well as other clinicopathological factors, including EGMA, *Helicobacter pylori* infection status, smoking status, alcohol consumption habit, body mass index (BMI), and proton pump inhibitor (PPI) or potassium‐competitive acid blocker (PCAB) use.

## 2 Methods

### 2.1 Study Design and Patient Selection

This retrospective cross‐sectional study was conducted at Shizuoka General Hospital. Consecutive Japanese patients with NADETs who underwent endoscopic or surgical resection between January 2015 and August 2021 were retrospectively identified.

Of 174 patients with 178 NADET lesions registered during the study period, patients were excluded because of inadequate visualization of the stomach and duodenum (*n* = 4), biopsy‐only diagnosis (*n* = 15), familial adenomatous polyposis (*n* = 5), history of upper gastrointestinal surgery (*n* = 7), unknown *H. pylori* infection status (*n* = 8), or unclassifiable mucin phenotype based on immunohistochemistry (*n* = 5). For patients with synchronous multiple NADETs, only the largest lesion was included. Finally, 130 patients with 130 lesions were analyzed (Figure 1).

**FIGURE 1:**
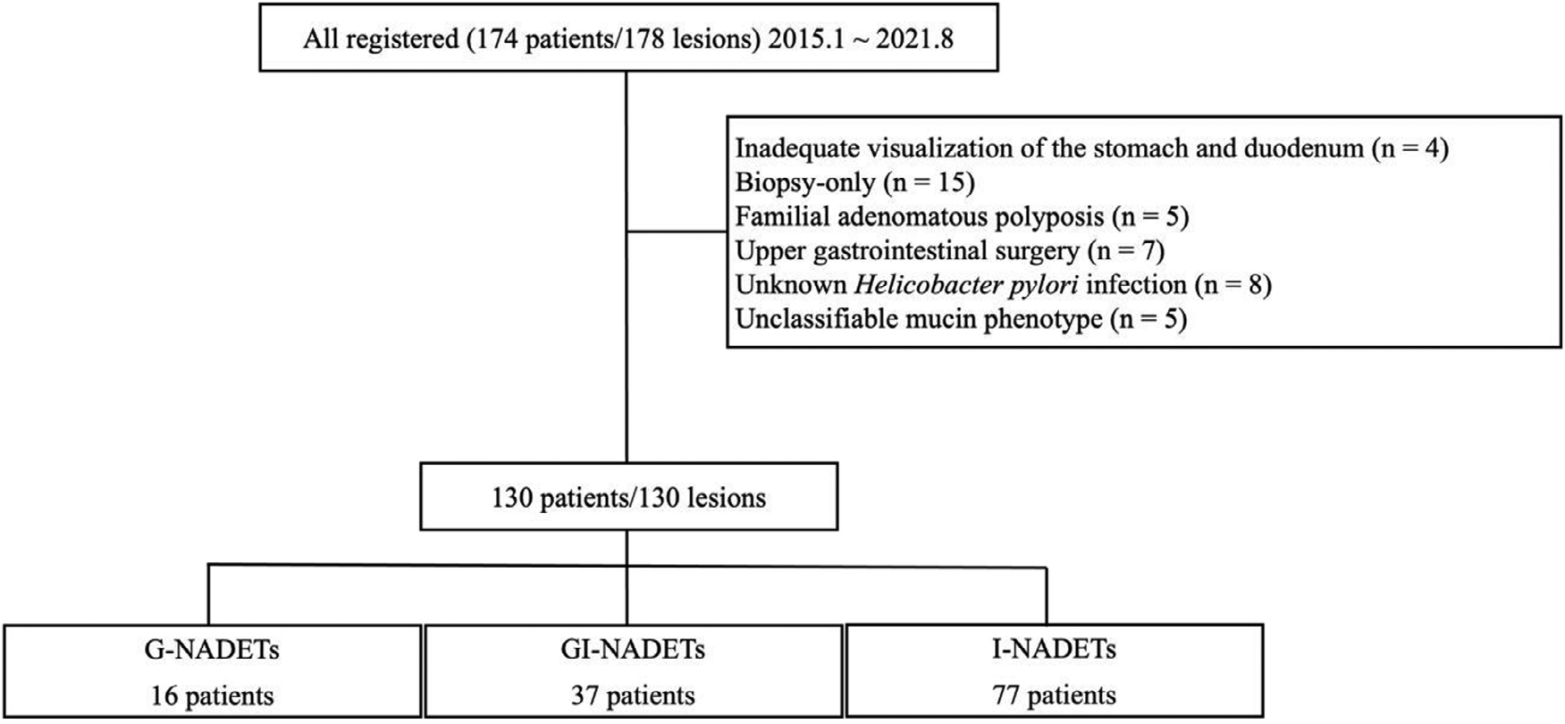
Patient flowchart. NADET, non‐ampullary duodenal epithelial tumor; G‐NADET, gastric‐type NADET; GI‐NADET, gastric–intestinal‐type NADET; I‐NADET, intestinal‐type NADET.

The primary objective was to determine the prevalence of concomitant G‐MLs according to NADET mucin phenotype. Secondary analyses were performed to evaluate associations between G‐ML presence and clinicopathological factors, including EGMA, *H. pylori* infection status, smoking status, alcohol consumption, BMI, and PPI or PCAB use.

### 2.2 Histopathological Evaluation and Mucin Phenotype Classification

NADETs were defined as duodenal epithelial neoplasms histologically diagnosed as tubular adenomas or adenocarcinomas. Macroscopic tumor morphology was assessed according to the Paris endoscopic classification [6]. Resected specimens were fixed in buffered formalin and sectioned at 2‐mm intervals (for endoscopic resection specimens) or 2‐ to 4‐mm intervals (for surgical specimens). Histopathological evaluation was performed by board‐certified gastrointestinal pathologists, and histological grade was classified according to the revised Vienna classification [7].

Mucin phenotype was determined by immunohistochemical staining using MUC5AC and MUC6 as gastric markers and MUC2 and CD10 as intestinal markers. Tumors were considered positive for each marker when > 10% of tumor cells showed positive staining. Tumors were classified as G‐NADETs when they were positive for gastric markers and negative for intestinal markers, gastric–intestinal‐type NADETs (GI‐NADETs) when they were positive for both gastric and intestinal markers, and intestinal‐type NADETs (I‐NADETs) when they were negative for gastric markers and positive for intestinal markers [8]. A representative case of a G‐NADET classified using this immunohistochemical approach is shown in Figure 2.

**FIGURE 2:**
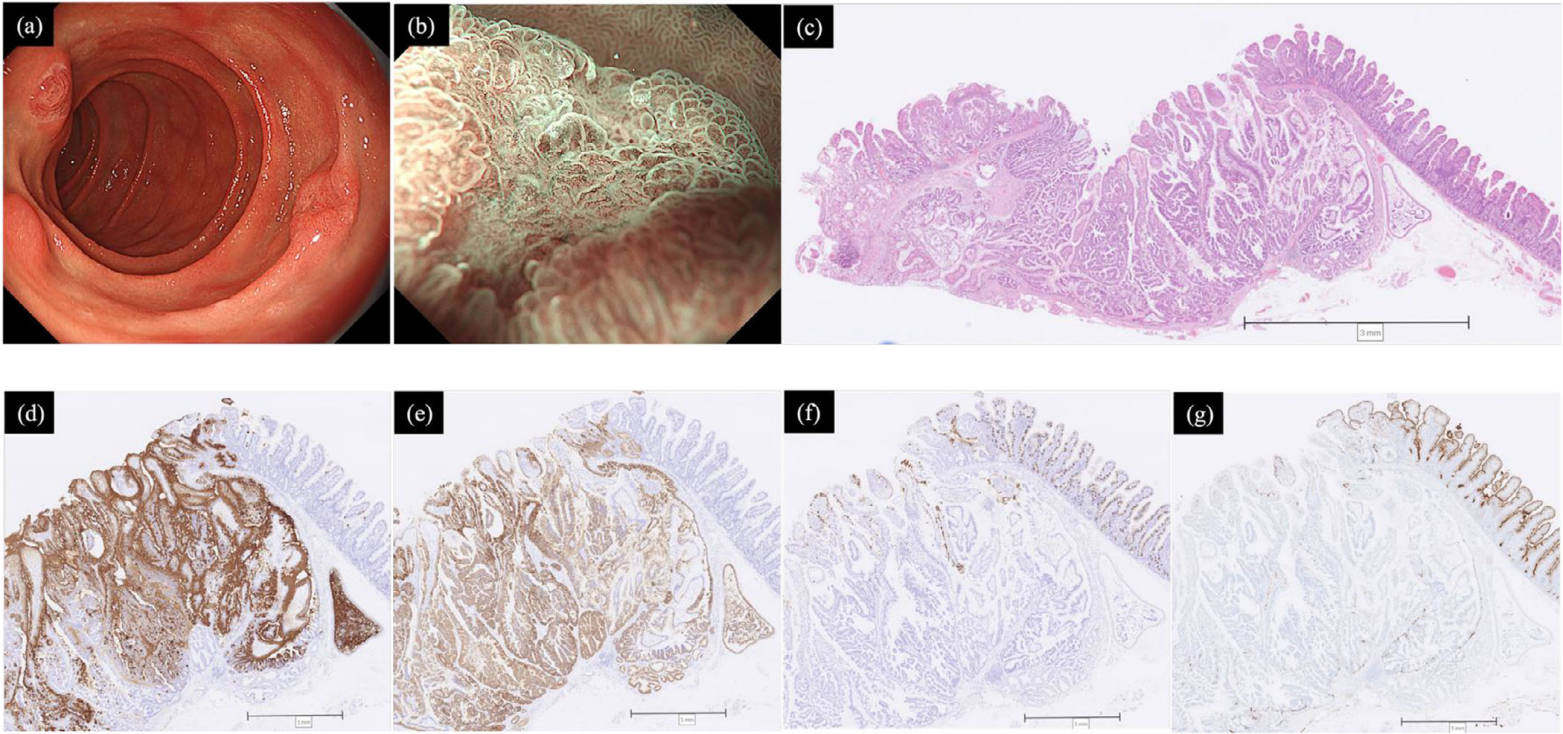
Representative endoscopic, histological, and immunohistochemical findings of a gastric‐type non‐ampullary duodenal epithelial tumor. (a) White‐light endoscopy showing a type 0‐IIc lesion on the side opposite the duodenal papilla. A concomitant gastric‐type mucosal lesion is present proximal to the papilla. (b) Magnifying narrow‐band imaging showing irregularly arranged gastric pit‐like structures of varying sizes within the depressed area. (c) Hematoxylin and eosin‐stained image showing a high‐grade epithelial neoplasm confined to the mucosa, corresponding to category 4.4 according to the revised Vienna classification. (d, e) Immunohistochemical staining positive for MUC5AC and MUC6. (f, g) Immunohistochemical staining negative for MUC2 and CD10.

### 2.3 Endoscopic Examination

Endoscopic examinations were performed using GIF‐H260Z, GIF‐H290Z, and GIF‐XZ1200 endoscopes with the EVIS LUCERA ELITE or EVIS X1 system (Olympus Corporation, Tokyo, Japan). All examinations were performed using systems capable of magnifying observation.

### 2.4 Definition and Assessment of G‐MLs

As described above, G‐MLs included gastric foveolar metaplasia, gastric heterotopia, and Brunner's gland hyperplasia. They were operationally defined as sporadic protruding duodenal lesions measuring ≥ 3 mm that were judged, based on endoscopic or histopathological findings, to correspond to these gastric‐type lesions. The 3‐mm cutoff was used to avoid overestimating G‐ML positivity by excluding trivial mucosal irregularities or physiological changes. For endoscopically diagnosed G‐MLs, recorded images were retrospectively reviewed by two board‐certified fellows of the Japan Gastroenterological Endoscopy Society, each with more than 20 years of endoscopic experience. Disagreements were resolved by a third board‐certified endoscopist.

As gastric foveolar metaplasia and gastric heterotopia are difficult to distinguish endoscopically, and Brunner's gland hyperplasia may accompany gastric foveolar metaplasia, endoscopically diagnosed G‐MLs were analyzed collectively without subtype classification. For histopathologically evaluated lesions, the subtype was recorded, and representative confirmed cases are shown in Figures 3, 4, 5.

**FIGURE 3:**
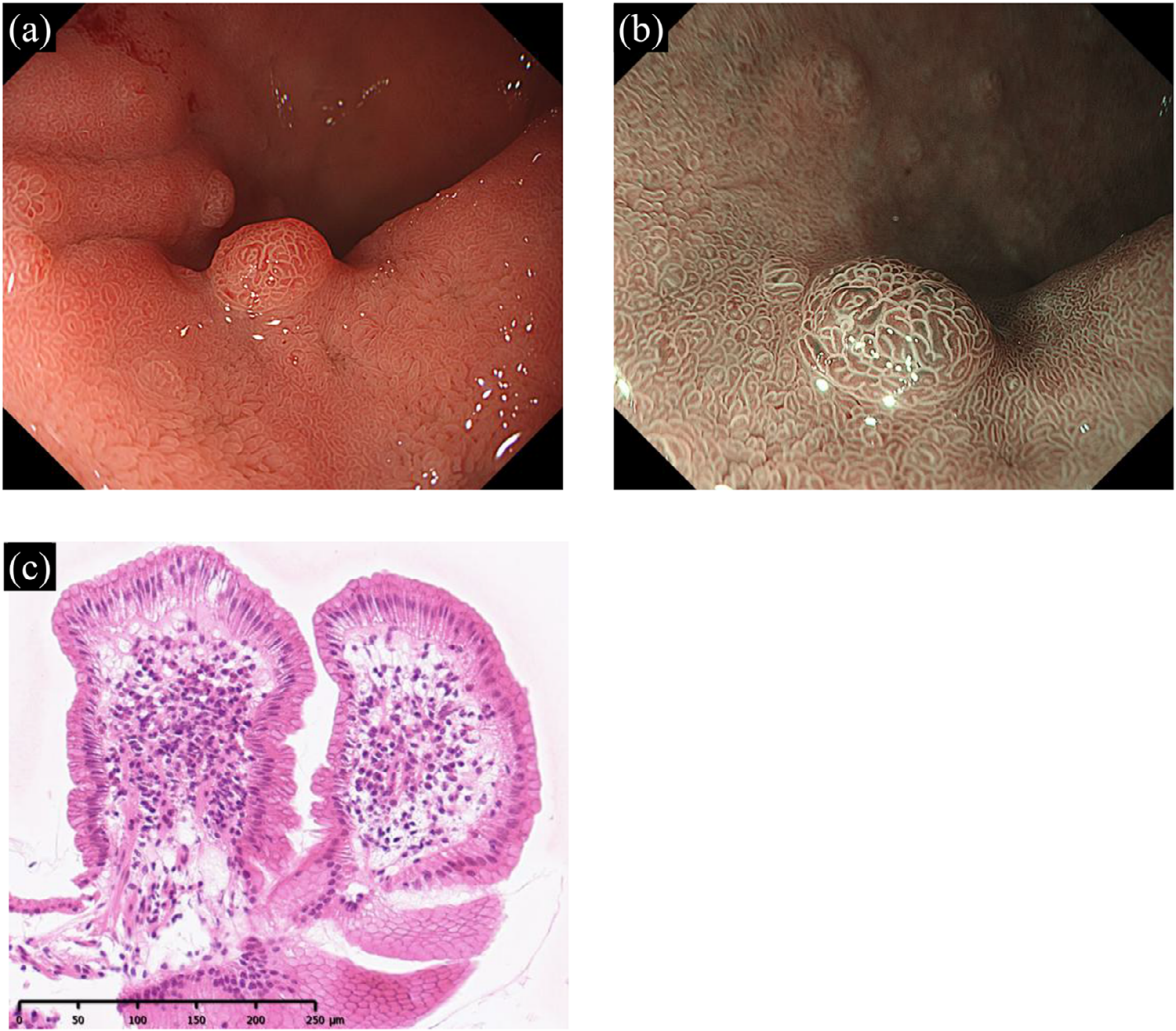
Representative endoscopic and histological findings of a gastric‐type mucosal lesion with gastric foveolar metaplasia. (a) White‐light endoscopic image showing a 3‐mm lesion in the duodenal bulb. (b) Magnifying narrow‐band imaging showing relatively regular gastric pit‐like surface structures. (c) Hematoxylin and eosin‐stained image of the biopsy specimen showing gastric foveolar metaplasia.

**FIGURE 4:**
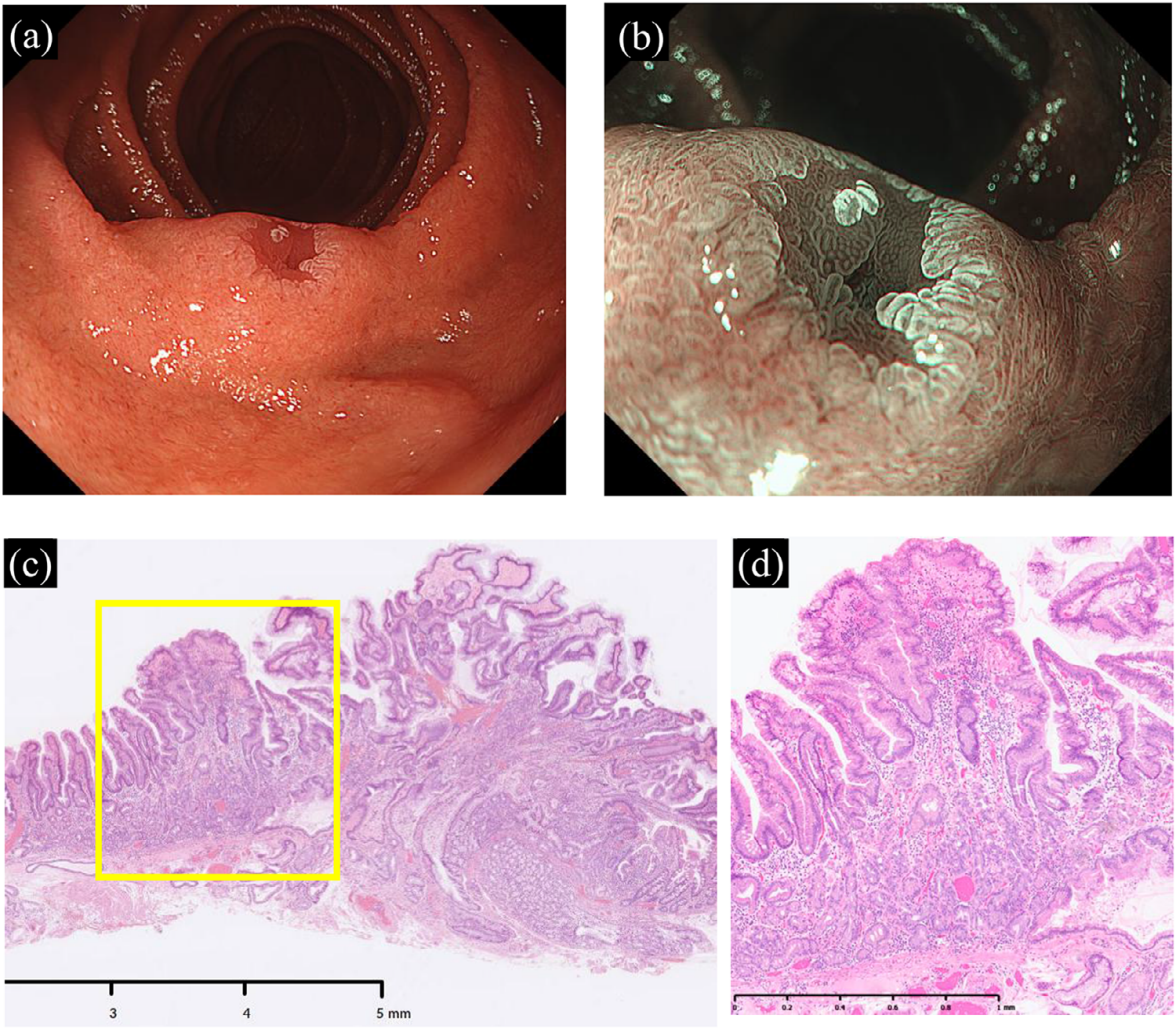
Representative endoscopic and histological findings of a gastric‐type mucosal lesion with gastric heterotopia. (a) White‐light endoscopic image showing an 8‐mm lesion on the oral side of the papilla in the descending duodenum. (b) Magnifying narrow‐band imaging showing relatively regular gastric pit‐like surface structures within the depressed area. (c) Hematoxylin and eosin‐stained image of the endoscopic mucosal resection specimen. (d) Close‐up view of the area outlined by the yellow rectangle in (c), showing heterotopic fundic glands accompanied by gastric foveolar metaplasia.

**FIGURE 5:**
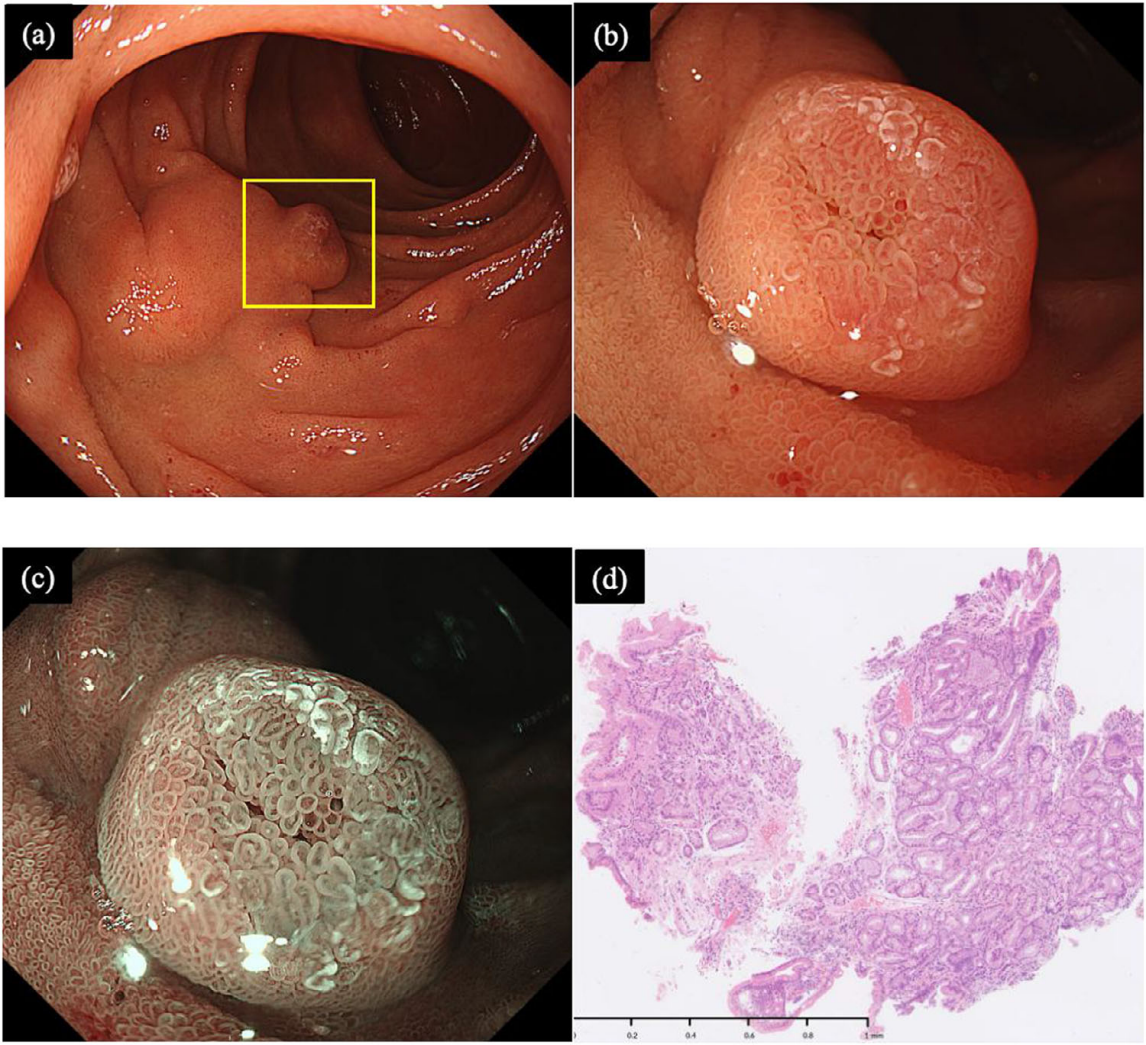
Representative endoscopic and histological findings of a gastric‐type mucosal lesion with Brunner's gland hyperplasia and gastric foveolar metaplasia. (a) White‐light endoscopic image showing a small protrusion on a submucosal tumor‐like elevation on the oral side of the papilla in the descending duodenum; the lesion is indicated with the yellow rectangle. (b) Close‐up view of the area outlined by the yellow rectangle in (a), showing regular gastric pit‐like surface structures at the top of the protrusion. (c) Narrow‐band imaging more clearly showing the regular gastric pit‐like surface structures. (d) Hematoxylin and eosin‐stained image of the biopsy specimen showing mucosal Brunner's gland hyperplasia with focal gastric foveolar metaplasia.

For each patient with G‐MLs, the number, location(s), representative or largest lesion size, macroscopic type, diagnostic basis, and available histological subtype of G‐MLs are summarized in Table S1. In patients with multiple G‐MLs, a histopathologically confirmed lesion was selected as representative when available; otherwise, the largest lesion was selected.

### 2.5 Evaluation of Gastric Environment and Clinical Variables

EGMA was assessed using the Kimura–Takemoto classification [9] and further categorized as closed‐type atrophy (C0–C3) or open‐type atrophy (O1–O3) according to the Kyoto classification of gastritis [10]. Closed‐type EGMA was regarded as mild EGMA.

*H. pylori* infection status was comprehensively determined based on serological antibody, urinary antibody, urea breath, and stool antigen tests, as well as endoscopic findings and documented eradication history. Serological anti‐*H. pylori* antibody was measured using *H. pylori*‐Latex SEIKEN (Denka Co., Ltd., Tokyo, Japan), with a positive cutoff value of ≥ 10 U/mL. Urinary anti‐*H. pylori* antibody was assessed using RAPIRUN *H. pylori* Antibody Stick (Eiken Chemical Co., Ltd., Tokyo, Japan), following the manufacturer's instructions. The urea breath test was performed using a POCone infrared spectrophotometer (Otsuka Electronics Co., Ltd., Osaka, Japan), with a positive cutoff value of Δ13C ≥ 2.5‰. The stool antigen test was performed using Testmate Pylori Antigen EIA (Wakamoto Pharmaceutical Co., Ltd., Tokyo, Japan), with a positive cutoff value of ≥ 0.100 by dual‐wavelength measurement at 450/630 nm.

Patients were classified as never infected, previously infected, or currently infected with *H. pylori*. Smoking status was defined according to the Centers for Disease Control and Prevention criteria [11]. Alcohol consumption status was categorized as drinking or nondrinking based on previously published definitions [12]. Obesity was defined as BMI ≥ 25 kg/m^2^ [13].

### 2.6 Statistical Analysis

Continuous variables are expressed as mean ± standard deviation or median (interquartile range), as appropriate, and categorical variables as number and percentage. Groups were compared using Student's *t*‐test for continuous variables and the chi‐square or Fisher's exact test for categorical variables. Fisher's exact test was used when any expected cell count was <5. Two‐sided *p*‐values < 0.05 indicated statistical significance. To explore potential confounding, logistic regression analyses were performed with G‐ML presence as the dependent variable and NADET mucin phenotype, categorized as G‐NADET versus non‐G‐NADET, as the primary explanatory variable. GI‐NADETs and I‐NADETs were combined as the reference group.

A location‐adjusted model was fitted, with NADET location categorized as the oral side of the papilla, including the duodenal bulb and descending portion oral to the papilla, versus the anal side of the papilla. Subsequently, an exploratory fully adjusted model including G‐NADET phenotype, NADET location, mild EGMA, smoking history, and current *H. pylori* infection was fitted. Mild EGMA was defined as C0–C3 according to the Kimura–Takemoto classification, smoking history as former/current smoker versus never smoked, and current *H. pylori* infection as currently infected with *H. pylori*.

Because of the small number of G‐NADET cases and sparse data, Firth‐penalized logistic regression was used to reduce small‐cohort bias and instability of parameter estimates [14]. Odds ratios (ORs) and 95% profile‐likelihood confidence intervals (CIs) were calculated. Sensitivity analysis was used to refit the fully adjusted model using a three‐category NADET location variable: the duodenal bulb, descending portion oral to the papilla, and descending portion anal to the papilla. All additional regression analyses were exploratory.

All statistical analyses were performed using EZR (Saitama Medical Center, Jichi Medical University, Saitama, Japan), a graphical user interface for R (The R Foundation for Statistical Computing, Vienna, Austria) [15].

## 3 Results

### 3.1 Study Population

The baseline clinicopathological characteristics of the patients are summarized in Table 1. Among the 130 lesions, 16 (12.3%) were classified as G‐NADETs, 37 (28.5%) as GI‐NADETs, and 77 (59.2%) as I‐NADETs.

**TABLE 1:** Baseline clinicopathological characteristics of patients with non‐ampullary duodenal epithelial tumors (NADETs) (*n* = 130).

Variable	Category or unit	Overall (*n* = 130)
Age	Years	66.3 ± 10.8
Sex	Female	39 (30.0)
	Male	91 (70.0)
Tumor location	Duodenal bulb	24 (18.5)
	Between the bulb and the papilla	60 (46.2)
	Distal to the papilla	46 (35.4)
Macroscopic type	0‐Is	27 (20.8)
	0‐IIa	69 (53.1)
	0‐IIc	32 (24.6)
	Advanced	2 (1.5)
Tumor maximum length	mm	8.5 (5.0–13.0)
Histology (Vienna classification)	Low‐grade neoplasia (category 3)	51 (39.2)
	High‐grade or invasive neoplasia (categories 4 and 5)	79 (60.8)
*Helicobacter pylori* status	Never infected	66 (50.8)
	Previously infected	36 (27.7)
	Currently infected	28 (21.5)
Smoking history	Yes	82 (63.1)
	No	48 (36.9)
Alcohol consumption	Yes	63 (48.5)
	No	67 (51.5)

Data are presented as mean ± standard deviation, median (interquartile range) for tumor maximum length, or number (percentage), as appropriate. Superficial lesions were classified according to the Paris classification, whereas advanced cancers were categorized separately. NADET, non‐ampullary duodenal epithelial tumor.

### 3.2 Prevalence and Characteristics of Concomitant G‐MLs

Concomitant G‐MLs were identified in 44 of 130 patients with NADETs (33.8%). Of these, 13 were histopathologically confirmed, whereas 31 were diagnosed based on endoscopic findings alone. Histopathological diagnoses were gastric heterotopia in one patient, gastric foveolar metaplasia in six, Brunner's gland hyperplasia in three, and Brunner's gland hyperplasia accompanied by gastric foveolar metaplasia in three.

Among the 44 patients with G‐MLs, the G‐MLs themselves were located in the duodenal bulb and/or descending portion oral to the papilla in 42 patients (95.5%) (Table S2).

### 3.3 Prevalence of G‐MLs According to NADET Mucin Phenotype

The prevalence of concomitant G‐MLs differed significantly according to NADET mucin phenotype. G‐MLs were observed in 13 of 16 G‐NADETs (81.3%), 10 of 37 GI‐NADETs (27.0%), and 21 of 77 I‐NADETs (27.3%) (*p* < 0.001). The prevalence was the highest among G‐NADETs (Figure 6).

**FIGURE 6:**
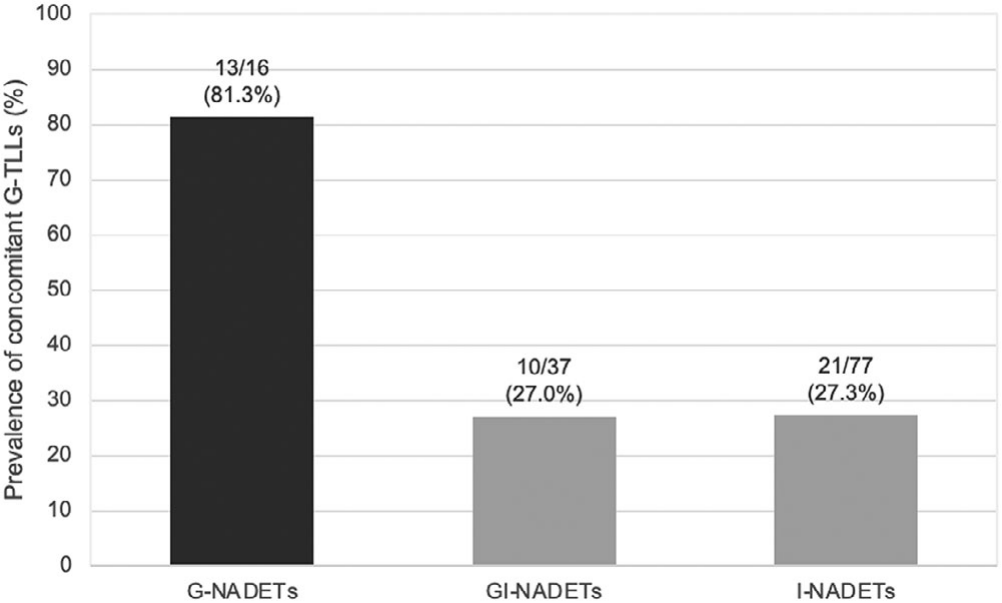
Prevalence of concomitant gastric‐type mucosal lesions according to the mucin phenotype of non‐ampullary duodenal epithelial tumors. Values above the bars indicate the number of G‐ML‐positive cases/total cases and the corresponding percentage. The prevalence of concomitant G‐MLs differed significantly according to the mucin phenotype of NADETs and was the highest in G‐NADETs (*p* < 0.001, Fisher's exact test). G‐ML, gastric‐type mucosal lesion; NADET, non‐ampullary duodenal epithelial tumor; G‐NADET, gastric‐type NADET; GI‐NADET, gastric–intestinal‐type NADET; I‐NADET, intestinal‐type NADET.

Most G‐NADETs were located on the oral side of the papilla (15/16; 93.8%). However, G‐ML prevalence was similar between NADETs located on the oral and anal sides of the papilla (29/84, 34.5% vs. 15/46, 32.6%) (Table S3).

In the unadjusted Firth‐penalized logistic regression analysis, G‐NADETs were associated with G‐ML presence (OR, 10.22; 95% CI, 3.23–41.76; *p* < 0.001). The association remained evident after adjustment for NADET location (adjusted OR, 11.44; 95% CI, 3.47–48.25; *p* < 0.001).

In the exploratory fully adjusted model including NADET location, mild EGMA, smoking history, and current *H. pylori* infection, G‐NADETs remained positively associated with G‐ML presence (adjusted OR, 7.00; 95% CI, 1.98–30.95; *p* = 0.002). The sensitivity analysis using the same covariates but representing NADET location as three anatomical categories yielded a consistent result (adjusted OR, 8.22; 95% CI, 1.98–45.66; *p* = 0.003) (Table S4).

### 3.4 Clinicopathological Factors Associated With G‐MLs

Clinicopathological factors associated with G‐ML presence are shown in Table 2. G‐ML presence was significantly associated with mild EGMA and smoking history, whereas current *H. pylori* infection was less frequent in patients with G‐MLs than in those without G‐MLs. Alcohol consumption, obesity, and PPI or PCAB use were not significantly associated with G‐ML presence.

**TABLE 2:** Clinicopathological factors according to the presence of concomitant gastric‐type mucosal lesions (G‐MLs).

Variable	G‐ML (+) (*n* = 44)	G‐ML (−) (*n* = 86)	*p*‐Value
Mild EGMA	40 (90.9)	64 (74.4)	0.026
Current *H. pylori* infection	4 (9.1)	24 (27.9)	0.014
Smoking history	34 (77.3)	48 (55.8)	0.016
Alcohol consumption	22 (50.0)	41 (47.7)	0.802
Obesity (BMI ≥ 25 kg/m^2^)	11 (25.0)	27 (31.4)	0.448
PPI or PCAB use	12 (27.3)	32 (37.2)	0.331

Data are presented as the number of patients (percentage). *p*‐Values were calculated using the chi‐square test or Fisher's exact test, as appropriate. Mild EGMA was defined as closed‐type atrophy (C0–C3) according to the Kimura–Takemoto classification. G‐ML, gastric‐type mucosal lesion; EGMA, endoscopic gastric mucosal atrophy; BMI, body mass index; PPI, proton pump inhibitor; PCAB, potassium‐competitive acid blocker; *H. pylori*, *Helicobacter pylori*.

## 4 Discussion

Concomitant G‐MLs were identified in approximately one‐third of patients with NADETs. G‐ML prevalence differed markedly according to NADET mucin phenotype and was the highest in patients with G‐NADETs. Additionally, G‐ML presence was associated with mild EGMA and smoking status and inversely associated with current *H. pylori* infection. These findings indicate an association between G‐MLs and G‐NADETs, but they should not be interpreted as evidence of a biological or precursor relationship.

The main finding of the present study was the higher prevalence of concomitant G‐MLs in patients with G‐NADETs than in those with GI‐NADETs or I‐NADETs. Matsubara et al. [4] reported activating mutations in GNAS and KRAS in gastric foveolar metaplasia, gastric heterotopia, and duodenal adenocarcinoma. Yamazaki et al. [16] reported a duodenal gastric foveolar–type hyperplastic polyp harboring GNAS and KRAS mutations. However, these pathological observations do not establish a biological relationship between G‐MLs and G‐NADETs. G‐MLs were observed in 81.3% of G‐NADETs, compared with 27.0% of GI‐NADETs and 27.3% of I‐NADETs. These results demonstrate an association but do not indicate that G‐MLs are precursor lesions or biologically related background lesions.

The association between G‐NADETs and G‐ML presence remained evident after adjustment for NADET location and in the exploratory fully adjusted model. Although both G‐NADETs and G‐MLs were concentrated in the proximal duodenum, G‐ML prevalence was similar between NADETs located on the oral and anal sides of the papilla, and the sensitivity analysis using three anatomical location categories yielded consistent results. Thus, NADET location alone is unlikely to fully explain the observed association. However, adjustment for anatomical location cannot account for differences in the extent or intensity of endoscopic observation. Therefore, the association may still be partly or entirely attributable to detection bias.

These findings should be interpreted cautiously. Only 16 G‐NADETs were included, CIs were wide, and residual confounding cannot be excluded. Because areas around the index tumor could have been examined and photographed more carefully than other duodenal areas, G‐MLs may have been preferentially detected near G‐NADETs, which were predominantly located on the oral side of the papilla. A standardized and systematic set of endoscopic images covering the entire duodenum was not available in all cases. Differences in endoscopic observation may partly or entirely explain the observed association, and adjustment for NADET location cannot eliminate this potential detection bias. As this study was retrospective and cross‐sectional, it cannot determine whether G‐MLs are precursor lesions, intermediary lesions, or associated background findings.

G‐ML presence was associated with mild EGMA and was less frequent in patients with current *H. pylori* infection. Mild EGMA may reflect relatively preserved gastric acid secretion, whereas current *H. pylori* infection and advanced atrophic gastritis are associated with reduced acid secretion [17, 18, 19]. However, gastric acid secretion was not directly measured, and the cross‐sectional design does not permit mechanistic interpretation of these associations. Figure S1 schematically summarizes the observed associations and is descriptive; it does not imply causal, precursor, or biological relationships.

Smoking was also associated with G‐ML presence. Previous studies have suggested that cigarette smoking can affect the gastroduodenal environment by influencing gastric acid secretion and impairing duodenal mucosal defense mechanisms, including acid‐stimulated bicarbonate secretion [20, 21]. Thus, smoking may contribute to the development or persistence of gastric‐type mucosal changes in the duodenum. However, causality between smoking and G‐MLs could not be determined. Further studies are needed to clarify the role of smoking and other environmental factors in G‐ML and G‐NADET development.

G‐MLs were analyzed collectively because reliable endoscopic differentiation among gastric foveolar metaplasia, gastric heterotopia, and Brunner's gland hyperplasia is difficult in routine practice. Brunner's gland hyperplasia may also be accompanied by gastric foveolar metaplasia, making strict subtype classification based on endoscopic findings alone unreliable. Therefore, endoscopically diagnosed lesions were not classified into individual subtypes; histopathological subtypes were recorded only when pathological evaluation was available. This approach was intended to improve the reproducibility of G‐ML assessment while acknowledging the limitations of retrospective endoscopic diagnosis.

This study has some limitations. First, it was retrospective and single‐center, with a relatively small cohort, particularly for G‐NADETs. Although Firth‐penalized logistic regression was used to reduce small‐cohort bias and instability of parameter estimates, the additional regression analyses were exploratory and should be interpreted cautiously. Second, G‐MLs were not histopathologically confirmed in all patients, and many were diagnosed by retrospective review of endoscopic images; therefore, misclassification could not be completely excluded. However, assessment was performed independently by experienced endoscopists, and histopathological subtypes were recorded when tissue specimens were available. Third, G‐ML detection may have been influenced by image quality, the extent of duodenal observation, and anatomical location. A standardized and systematic set of endoscopic images covering the entire duodenum was not available in all cases, and adjustment for NADET location could not fully eliminate this potential detection bias. Fourth, as gastric acid secretion was not directly measured, the relationship among mild EGMA, current *H. pylori* infection, G‐MLs, and gastric acid exposure remains hypothetical. Finally, molecular analyses of G‐MLs and NADETs were not performed. Prospective studies incorporating standardized duodenal observation, histopathological confirmation, gastric acid assessment, and molecular analysis are needed to clarify the association between G‐MLs and G‐NADETs.

In conclusion, concomitant G‐MLs occurred in approximately one‐third of patients with NADETs and were most frequent in patients with G‐NADETs. G‐MLs were associated with mild EGMA and smoking history and were inversely associated with current *H. pylori* infection. An association between G‐NADETs and G‐ML presence remained evident after exploratory adjustment for NADET location and other clinicopathological factors. However, the cross‐sectional design, non‐standardized endoscopic observation, and potential detection bias preclude conclusions regarding a biological relationship. Prospective studies using standardized and systematic observation of the entire duodenum are required to confirm this association.

## Author Contributions

**Kazuya Ohno**: conceptualization, methodology, data curation, formal analysis, investigation, and writing – original draft. **Eiji Nakatani**: methodology, formal analysis, statistical analysis, and writing – review and editing. **Takafumi Kurokami**, **Asami Kawai**, **Masanori Matsuda**, **Yuichi Masui**, **Tatsunori Satoh**, **Taiyo Hirata**, **Shodai Takeda**, **Yoshihiro Uehara**, **Kodai Baba**, **Yuka Ozaki**, **Tomoki Hikichi**, **Haruna Takahashi**, **Koki Sakaguchi**, **Kazuhito Honda**, **Tomomi Maeda**, **Moeri Hotta**, and **Naofumi Nakamura**: investigation and data curation. **Makoto Suzuki**: pathological evaluation and validation. **Ken Haruma**: supervision and writing – review and editing. All authors contributed to the interpretation of the data and approved the final version of the manuscript.

## Funding

The authors have nothing to report.

## Ethics Statement

This study was conducted in accordance with the principles of the Declaration of Helsinki.

## Consent

The requirement for written informed consent was waived owing to the retrospective nature of the study. Instead, an opt‐out method was used, and information about the study was disclosed on the institutional website.

## Conflicts of Interest

The authors declare no conflicts of interest.

Approval of the research protocol by an Institutional Review Board: This study was approved by the Ethics Committee of Shizuoka General Hospital (approval number: SGHIRB #2024059).

## Supporting information



Supporting File: 1 deo270443‐sup‐0001‐TableS1.docx

Supporting File: 2 deo270443‐sup‐0002‐TableS2.docx

Supporting File: 3 deo270443‐sup‐0004‐TableS3.docx

Supporting File: 4 deo270443‐sup‐0004‐TableS4.docx

Supporting File: 5 deo270443‐sup‐0005‐FigureS1.docx

## Data Availability

The data generated/analyzed in this study are available from the corresponding author on reasonable request.

## References

[1] K. Okada , J. Fujisaki , A. Kasuga , et al., “Sporadic Nonampullary Duodenal Adenoma in the Natural History of Duodenal Cancer: A Study of Follow‐Up Surveillance,” American Journal of Gastroenterology 106, no. 2 (2011): 357–364, 10.1038/ajg.2010.422. 21139577

[2] T. Ushiku , T. Arnason , M. Fukayama , and G. Y. Lauwers , “Extra‐Ampullary Duodenal Adenocarcinoma,” American Journal of Surgical Pathology 38, no. 11 (2014): 1484–1493, 10.1097/PAS.0000000000000278. 25310836

[3] R. Kushima , H. J. Rüthlein , M. Stolte , M. Bamba , T. Hattori , and F. Borchard , “‘Pyloric Gland‐Type Adenoma’ Arising in Heterotopic Gastric Mucosa of the Duodenum, With Dysplastic Progression of the Gastric Type,” Virchows Archiv 435, no. 4 (1999): 452–457, 10.1007/s004280050425. 10526011

[4] A. Matsubara , R. Ogawa , H. Suzuki , et al., “Activating GNAS and KRAS Mutations in Gastric Foveolar Metaplasia, Gastric Heterotopia, and Adenocarcinoma of the Duodenum,” British Journal of Cancer 112, no. 8 (2015): 1398–1404, 10.1038/bjc.2015.104. PMC4402452 25867268

[5] K. Ohno , E. Nakatani , T. Kurokami , et al., “Relationship between Gastric Mucosal Atrophy by Endoscopy and Non‐ampullary Duodenal Epithelial Tumors,” World Journal of Gastrointestinal Oncology 17, no. 2 (2025): 100545, 10.4251/wjgo.v17.i2.100545. PMC11756005 39958533

[6] Participants in the Paris Workshop , “The Paris Endoscopic Classification of Superficial Neoplastic Lesions: Esophagus, Stomach, and Colon: November 30 to December 1, 2002,” Gastrointestinal Endoscopy 58, no. 6(suppl) (2003): S3–S43, 10.1016/s0016-5107(03)02159-x. 14652541

[7] M. F. Dixon , “Gastrointestinal Epithelial Neoplasia: Vienna Revisited,” Gut 51, no. 1 (2002): 130–131, 10.1136/gut.51.1.130. PMC1773259 12077106

[8] Y. Toya , M. Endo , S. Yamada , et al., “The Mucin Phenotype Does Not Affect the Endoscopic Resection Outcome of Non‐ampullary Duodenal Epithelial Tumors,” Endoscopy International Open 9, no. 9 (2021): E1297–E1302, 10.1055/a-1477-3186. PMC8367432 34466350

[9] K. Kimura and T. Takemoto , “An Endoscopic Recognition of the Atrophic Border and Its Significance in Chronic Gastritis,” Endoscopy 1, no. 3 (1969): 87–97, 10.1055/s-0028-1098086.

[10] O. Toyoshima , T. Nishizawa , and K. Koike , “Endoscopic Kyoto Classification of *Helicobacter pylori* Infection and Gastric Cancer Risk Diagnosis,” World Journal of Gastroenterology 26, no. 5 (2020): 466–477, 10.3748/wjg.v26.i5.466. PMC7015719 32089624

[11] Centers for Disease Control and Prevention (CDC) , “Vital Signs: Current Cigarette Smoking Among Adults Aged ≥ 18 Years—United States, 2005–2010,” MMWR Morbidity & Mortality Weekly Report 60, no. 35 (2011): 1207–1212. 21900875

[12] B. S. San José , J. A. van Oers , H. van de Mheen , H. F. Garretsen , and J. P. Mackenbach , “Drinking Patterns and Health Outcomes: Occasional versus Regular Drinking,” Addiction 95, no. 6 (2000): 865–872, 10.1080/09652140050031617. 10946436

[13] H. B. Daher and A. I. Sharara , “Gastroesophageal Reflux Disease, Obesity and Laparoscopic Sleeve Gastrectomy: The Burning Questions,” World Journal of Gastroenterology 25, no. 33 (2019): 4805–4813, 10.3748/wjg.v25.i33.4805. PMC6737315 31543675

[14] G. Heinze and M. Schemper , “A Solution to the Problem of Separation in Logistic Regression,” Statistics in Medicine 21, no. 16 (2002): 2409–2419, 10.1002/sim.1047. 12210625

[15] Y. Kanda , “Investigation of the Freely Available Easy‐to‐Use Software EZR for Medical Statistics,” Bone Marrow Transplantation 48, no. 3 (2013): 452–458, 10.1038/bmt.2012.244. PMC3590441 23208313

[16] K. Yamazaki , R. Kushima , N. Ozawa , et al., “Gastric Foveolar‐Type Hyperplastic Polyp of the Duodenum With GNAS and KRAS Mutations: A Potential Precursor to Neoplasia,” DEN Open 6, no. 1 (2026): e70207, 10.1002/deo2.70207. PMC12436171 40964650

[17] P. Sipponen and H. Hyvärinen , “Role of *Helicobacter pylori* in the Pathogenesis of Gastritis, Peptic Ulcer and Gastric Cancer,” Scandinavian Journal of Gastroenterology Supplement 196 (1993): 3–6, 10.3109/00365529309098333. 8341988

[18] K. Haruma , T. Kamada , H. Kawaguchi , et al., “Effect of Age and *Helicobacter pylori* Infection on Gastric Acid Secretion,” Journal of Gastroenterology & Hepatology 15, no. 3 (2000): 277–283, 10.1046/j.1440-1746.2000.02131.x. 10764028

[19] Y. Liu , N. Uemura , S. D. Xiao , G. N. J. Tytgat , and F. J. W. T. Kate , “Agreement Between Endoscopic and Histological Gastric Atrophy Scores,” Journal of Gastroenterology 40, no. 2 (2005): 123–127, 10.1007/s00535-004-1511-x. 15770394

[20] M. A. Ainsworth , D. L. Hogan , M. A. Koss , and J. I. Isenberg , “Cigarette Smoking Inhibits Acid‐Stimulated Duodenal Mucosal Bicarbonate Secretion,” Annals of Internal Medicine 119, no. 9 (1993): 882–886, 10.7326/0003-4819-119-9-199311010-00003. 8214999

[21] D. R. Fletcher , A. Shulkes , and K. J. Hardy , “The Effect of Cigarette Smoking on Gastric Acid Secretion and Gastric Mucosal Blood Flow in Man,” Australian & New Zealand Journal of Medicine 15, no. 4 (1985): 417–420, 10.1111/j.1445-5994.1985.tb02763.x. 3866537

