# Supplementary material for: Prevalence and Clinical Significance of Gastric‐Type Mucosal Lesions in Non‐Ampullary Duodenal Epithelial Tumors Based on Mucin Phenotype
Source: DEN Open. 2026 Oct 26;7(1):e70443. doi: 10.1002/deo2.70443 (PMC13710890; doi:10.1002/deo2.70443)
Supplement: Supplementary file 1 [file DEO2-7-e70443-s001.docx]

**Supplementary Table 2. Anatomical location of G-MLs among 44 G-ML-positive patients.**

| **G-ML location** | **Patients, n (%)** |
| --- | --- |
| Duodenal bulb only | 17 (38.6) |
| Descending portion oral to the papilla only | 16 (36.4) |
| Both duodenal bulb and descending portion oral to the papilla | 9 (20.5) |
| Descending portion anal to the papilla only | 2 (4.5) |
| Any proximal location (bulb and/or descending portion oral to the papilla) | 42 (95.5) |

This table shows the anatomical distribution of G-MLs themselves among patients with concomitant G-MLs. The proximal duodenum was defined as the duodenal bulb and/or the descending portion oral to the papilla. G-ML, gastric-type mucosal lesion.
