# Supplementary material for: Prevalence and Clinical Significance of Gastric‐Type Mucosal Lesions in Non‐Ampullary Duodenal Epithelial Tumors Based on Mucin Phenotype
Source: DEN Open. 2026 Oct 26;7(1):e70443. doi: 10.1002/deo2.70443 (PMC13710890; doi:10.1002/deo2.70443)
Supplement: Supplementary file 2 [file DEO2-7-e70443-s002.docx]

**Supplementary Table 3. Distribution of NADET location according to G-NADET phenotype and G-ML status.**

| **NADET location** | **All NADETs, n (% of 130)** | **G-NADETs, n (% of 16)** | **NADET location among patients with G-MLs,  n (% of 44)** | **G-ML prevalence, n/N (%)** |
| --- | --- | --- | --- | --- |
| Duodenal bulb | 24 (18.5) | 11 (68.8) | 11 (25.0) | 11/24 (45.8) |
| Descending portion oral to the papilla | 60 (46.2) | 4 (25.0) | 18 (40.9) | 18/60 (30.0) |
| Descending portion anal to the papilla | 46 (35.4) | 1 (6.2) | 15 (34.1) | 15/46 (32.6) |
| Oral side of the papilla | 84 (64.6) | 15 (93.8) | 29 (65.9) | 29/84 (34.5) |

Values in the second to fourth columns are column percentages. The denominator was 130 for all NADETs, 16 for G-NADETs, and 44 for G-ML-positive cases. The fourth column shows the distribution of NADET location among patients with G-MLs and does not represent the anatomical location of the G-ML itself. G-ML prevalence by NADET location is shown as the number of G-ML-positive cases/total cases and the corresponding percentage. The oral side of the papilla includes the duodenal bulb and the descending portion oral to the papilla. NADET, non-ampullary duodenal epithelial tumor; G-NADET, gastric-type NADET; G-ML, gastric-type mucosal lesion.
