# Supplementary material for: Prevalence and Clinical Significance of Gastric‐Type Mucosal Lesions in Non‐Ampullary Duodenal Epithelial Tumors Based on Mucin Phenotype
Source: DEN Open. 2026 Oct 26;7(1):e70443. doi: 10.1002/deo2.70443 (PMC13710890; doi:10.1002/deo2.70443)
Supplement: Supplementary file 3 [file DEO2-7-e70443-s003.docx]

Supplementary Figure 1. Schematic summary of the associations observed among G-MLs, G-NADETs, mild EGMA, current Helicobacter pylori infection, and smoking history. This schematic summarizes the associations observed in the present cross-sectional study and does not indicate causal, precursor, or biological relationships. Potential detection bias resulting from non-standardized endoscopic observation should also be considered. G-MLs, gastric-type mucosal lesions; G-NADETs, gastric-type non-ampullary duodenal epithelial tumors; EGMA, endoscopic gastric mucosal atrophy; H. pylori, Helicobacter pylori.
