# Supplementary material for: Prevalence and Clinical Significance of Gastric‐Type Mucosal Lesions in Non‐Ampullary Duodenal Epithelial Tumors Based on Mucin Phenotype
Source: DEN Open. 2026 Oct 26;7(1):e70443. doi: 10.1002/deo2.70443 (PMC13710890; doi:10.1002/deo2.70443)
Supplement: Supplementary file 4 [file DEO2-7-e70443-s004.docx]

**Supplementary Table 1. Detailed characteristics of concomitant gastric-type mucosal lesions.**

| Case No. | Multiplicity of G-MLs | Location | Representative/largest size (mm) | Gross type | Diagnostic basis | Histological subtype/finding |
| --- | --- | --- | --- | --- | --- | --- |
| 1 | Multiple (≥2) | D2 oral | 12 | 0-Is | Histological | Gastric heterotopia |
| 2 | Single | D2 oral | 5 | 0-IIa | Histological | Brunner’s gland hyperplasia |
| 3 | Multiple (≥2) | Bulb, D2 oral | 12 | 0-Is | Histological | Gastric foveolar metaplasia with Brunner’s gland hyperplasia |
| 4 | Multiple (≥2) | Bulb | 7 | 0-IIa | Histological | Gastric foveolar metaplasia |
| 5 | Multiple (≥2) | Bulb, D2 oral | 7 | 0-IIa | Histological | Gastric foveolar metaplasia with Brunner’s gland hyperplasia |
| 6 | Multiple (≥2) | Bulb, D2 oral | 8 | 0-IIa | Histological | Gastric foveolar metaplasia |
| 7 | Multiple (≥2) | D2 oral | 10 | 0-IIa | Histological | Gastric foveolar metaplasia |
| 8 | Single | D2 oral | 6 | 0-Is | Histological | Gastric foveolar metaplasia |
| 9 | Single | Bulb | 7 | 0-IIa | Histological | Brunner’s gland hyperplasia |
| 10 | Multiple (≥2) | Bulb, D2 oral | 6 | 0-Is | Histological | Gastric foveolar metaplasia |
| 11 | Single | D2 anal | 10 | 0-Is | Histological | Gastric foveolar metaplasia with Brunner’s gland hyperplasia |
| 12 | Single | Bulb | 5 | 0-IIa | Histological | Gastric foveolar metaplasia |
| 13 | Single | D2 oral | 7 | 0-Is | Histological | Brunner’s gland hyperplasia |
| 14 | Single | D2 oral | 6 | 0-IIa | Endoscopic | – |
| 15 | Single | Bulb | 5 | 0-Is | Endoscopic | – |
| 16 | Single | D2 oral | 5 | 0-Is | Endoscopic | – |
| 17 | Single | D2 oral | 4 | 0-IIa | Endoscopic | – |
| 18 | Multiple (≥2) | D2 oral | 10 | 0-IIa | Endoscopic | – |
| 19 | Single | D2 oral | 8 | 0-IIa | Endoscopic | – |
| 20 | Single | Bulb | 12 | 0-Is | Endoscopic | – |
| 21 | Single | Bulb | 6 | 0-IIa | Endoscopic | – |
| 22 | Single | Bulb | 4 | 0-IIa | Endoscopic | – |
| 23 | Multiple (≥2) | Bulb, D2 oral | 5 | 0-IIa | Endoscopic | – |
| 24 | Multiple (≥2) | D2 oral | 10 | 0-IIa | Endoscopic | – |
| 25 | Single | Bulb | 5 | 0-IIa | Endoscopic | – |
| 26 | Multiple (≥2) | D2 oral | 3 | 0-Is | Endoscopic | – |
| 27 | Single | Bulb | 3 | 0-IIa | Endoscopic | – |
| 28 | Single | D2 oral | 7 | 0-IIa | Endoscopic | – |
| 29 | Single | Bulb | 6 | 0-IIa | Endoscopic | – |
| 30 | Single | D2 anal | 5 | 0-Is | Endoscopic | – |
| 31 | Multiple (≥2) | Bulb, D2 oral | 10 | 0-IIa | Endoscopic | – |
| 32 | Multiple (≥2) | Bulb | 3 | 0-IIa | Endoscopic | – |
| 33 | Single | Bulb | 3 | 0-IIa | Endoscopic | – |
| 34 | Multiple (≥2) | Bulb | 5 | 0-IIa | Endoscopic | – |
| 35 | Multiple (≥2) | D2 oral | 7 | 0-Is | Endoscopic | – |
| 36 | Single | Bulb | 5 | 0-IIa | Endoscopic | – |
| 37 | Multiple (≥2) | Bulb | 7 | 0-IIa | Endoscopic | – |
| 38 | Single | D2 oral | 4 | 0-IIa | Endoscopic | – |
| 39 | Single | Bulb | 4 | 0-Is | Endoscopic | – |
| 40 | Single | D2 oral | 5 | 0-IIa | Endoscopic | – |
| 41 | Multiple (≥2) | Bulb, D2 oral | 7 | 0-Is | Endoscopic | – |
| 42 | Multiple (≥2) | Bulb, D2 oral | 5 | 0-IIa | Endoscopic | – |
| 43 | Single | Bulb | 6 | 0-Is | Endoscopic | – |
| 44 | Multiple (≥2) | Bulb, D2 oral | 7 | 0-Is | Endoscopic | – |

This table summarizes the number, location, representative or largest lesion size, macroscopic type, diagnostic basis, and histopathological subtype of concomitant G-TLLs. In patients with multiple G-MLs, a histopathologically confirmed lesion was selected as the representative lesion when available; otherwise, the largest lesion was selected. G-TLL, gastric-type mucosal lesion; GH, gastric heterotopia; GFM, gastric foveolar metaplasia; BGH, Brunner’s gland hyperplasia; NADET, non-ampullary duodenal epithelial tumor. D2 oral, descending portion oral to the papilla; D2 anal, descending portion anal to the papilla.
