# Supplementary material for: Prevalence and Clinical Significance of Gastric‐Type Mucosal Lesions in Non‐Ampullary Duodenal Epithelial Tumors Based on Mucin Phenotype
Source: DEN Open. 2026 Oct 26;7(1):e70443. doi: 10.1002/deo2.70443 (PMC13710890; doi:10.1002/deo2.70443)
Supplement: Supplementary file 5 [file DEO2-7-e70443-s005.docx]

**Supplementary Table 4. Association between G-NADET phenotype and the presence of G-MLs in Firth penalized logistic regression analyses**

| Model | OR | 95% CI | P value |
| --- | --- | --- | --- |
| Unadjusted model | 10.22 | 3.23–41.76 | <0.001 |
| Adjusted for NADET location | 11.44 | 3.47–48.25 | <0.001 |
| Fully adjusted model | 7.00 | 1.98–30.95 | 0.002 |
| Sensitivity analysis using three location categories | 8.22 | 1.98–45.66 | 0.003 |

The dependent variable was G-ML presence, and the primary explanatory variable was G-NADET phenotype. The location-adjusted model included binary NADET location, categorized as the oral versus anal side of the papilla. The fully adjusted model additionally included mild endoscopic gastric mucosal atrophy, smoking history, and current *Helicobacter pylori* infection. The sensitivity analysis included the same covariates as the fully adjusted model but represented NADET location using three anatomical categories: the duodenal bulb, the descending portion oral to the papilla, and the descending portion anal to the papilla. OR, odds ratio; CI, confidence interval; NADET, non-ampullary duodenal epithelial tumor; G-NADET, gastric-type NADET; G-ML, gastric-type mucosal lesion.
